# Advanced adiabatic compressed air energy storage systems dynamic modelling: Impact of the heat storage device

**DOI:** 10.1016/j.heliyon.2024.e40730

**Published:** 2024-11-27

**Authors:** Victor Dupin, David Teixeira

**Affiliations:** IFP Energies nouvelles, 1 et 4 avenue de Bois Préau, 92852, Rueil-Malmaison, France

## Abstract

Advanced Adiabatic Compressed Air Energy Storage (AACAES) is a technology for storing energy in thermomechanical form. This technology involves several equipment such as compressors, turbines, heat storage capacities, air coolers, caverns, etc. During charging or discharging, the heat storage and especially the cavern will induce transient behavior of operating points, notably temperature, pressure, and volume flow. In contrast, the optimal use of turbomachinery requires it to be as stationary as possible. AACAES technology therefore requires transient modelling to optimize its design. This paper presents a modular and adaptable numerical tool capable of simulating the dynamic behavior of different thermomechanical storage systems. This tool is then applied to an AACAES system to analyze the impact of various configurations on the variability of volume flow. The aim is to optimize Round Trip Efficiency (RTE) while limiting the maximum variation in volume flow. First, the effect of the size of fixed-bed Thermal Energy Storage (TES) is analyzed. Then, we analyze the behavior of the system with heat management based on a heat exchanger and a heat transfer fluid. In addition, the effect of adding pressure control at the cavern inlet/outlet on the two previous variants is studied.

## 1 Introduction

Renewable energy has seen accelerated growth in recent years, especially solar and wind power [1]. However, because both sun and wind are not controllable, flexibility is required to match electricity supply and demand at all times and maintain the integrity of the grid. There are several sources of flexibility to meet this challenge: generation plants on the supply side, peak cuts off on the demand‐side, and energy storage acting on both [1]. Among those, generation plants are the most used, but they have to be limited since they are mostly working with fossil fuels. Peak cut off is usually a good solution, but its effect may be limited in terms of capacity, depending on the power demand at the time of the peak. Finally, storage is flexible, making it possible to switch from periods of surplus production to periods of deficit [2].

Each electrical storage system is designed for a specific application [3]. Typically, integrating renewable energy into the grid would require couple of hours of storage [[3], [4], [5]], for example, to compensate for daily fluctuations in photovoltaic production [6]. Among the electricity storage systems for such application, Pumped Hydro Storage (PHS) is by far the most common and the most mature technology [7]. But, the major drawback of those systems lies on the scarcity of appropriate sites [7,8]. Massive battery storage is newer, and although they are known for their high efficiency, their short response time and energy density [2,9], few are already operating, mainly because of cost.

Finally, less mature are the thermomechanical processes that are still in their infancy and for which much research is needed to fully assess their potential. For example, there are two large scale Compressed Air Energy Storage (CAES) units in the world. The first, in Huntorf, Germany operating since 1978 which can generate 290 MW for 2 h and the second, in McIntosh, Alabama, USA operating since 1991 with a 110 MW capacity up to 26 h. Both compress air into big caves and store the energy mainly under pressure form, *ie* potential form. The discharging is made through turbines. These two examples, which have been in operation for years, have shown that CAES is relevant but, because of the large temperature drop occurring during the discharge, CAES relies on conventional gas turbine generation to heat the air back while expanding it [8]. This requirement of fossil fuel makes it less attractive.

**Fig. 1:**
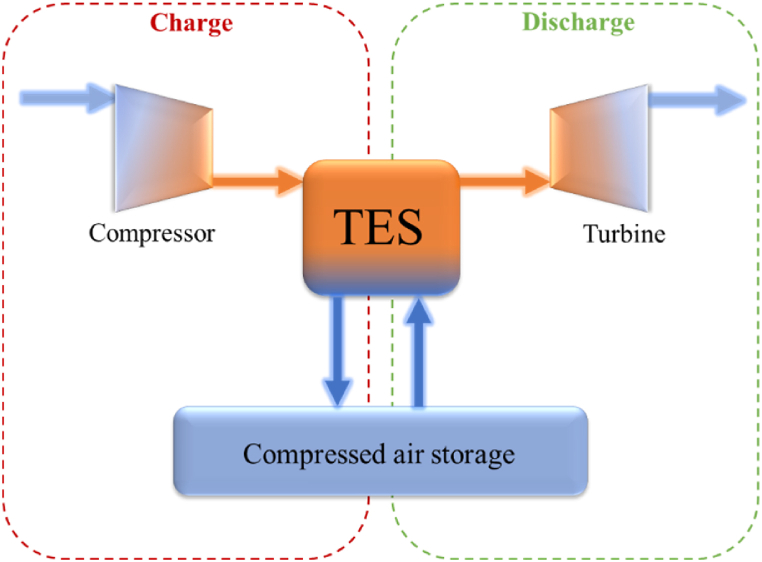
Schematic drawing of AACAES principle.

To overcome with this, Advanced Adiabatic Compressed Air Energy Storage (AACAES) can do without burning gas as it stores the heat generated by the compression so that it can be returned during discharging phase [10,11](Fig. 1). This technology is much less mature and only two large scale unit are operating, in China: a 100MW/400 MWh plant in Zhangjiakou launched in October 2022 and a 300MW/1.8 GWh in Feicheng city connected to the grid in June 2024. Hydrostor claims to develop some [10,12,13] up to 500 MW capacity in North America. And, in Europe, there was only one notable small-scale project in 2016 [12] from the ALACAES company in Switzerland. There are many possible variants of AACAES still under investigation: those using conventional turbomachinery or isothermal compression/expansion cycles, those storing the air at constant pressure etc [11]. Each time with the aim of optimizing the Round Trip Efficiency (RTE) or limiting the costs of turbomachinery, the cavern, and heat storage systems, etc. This paper will focus on the latter.

In an AACAES process, the heat is stored via thermal energy storage systems (TES) that can be of two types [10]: those using indirect-contact heat exchanger and a separate thermal fluid and those using direct air contact, such as packed bed columns for example. The latter, which is discussed later in this paper, has the advantage of being able to offer high rates of heat transfer and withstand higher temperatures and pressures.

In practice, compressing air from atmospheric pressure to its storage pressure around 80–150 bars, implies several stages with several compressors, expanders, and inter-cooling between each. The complexity of these AACAES processes, which require a large amount of equipment, means that they usually operate under transient conditions.

For example, as the cavern is filled or emptied, the inlet or outlet pressure changes, generating volume flow variations. Or, in the case of direct contact heat exchange the outlet temperature from the TES may vary considerably as it reaches its storage limit causing significant volume flow variations. In addition to this, the need to control the output power of compressors and turbines, may require adjustments to mass flow and therefore reduced flow.

Transient behavior, and in particular volume flow variation has implications for turbomachinery, for example, because each turbine or compressor is designed for a specific application and is optimally controlled around a reduced flow rate level that should vary little, otherwise their efficiency can drop drastically. Furthermore, Luo et al., in his optimization paper [14], showed that turbomachinery efficiency is one of the most important parameters for the Round Trip Efficiency (RTE) calculation, defined as the ratio between the electrical energy restored during discharging and the electrical energy consumed during charging.

Therefore, it is essential to be able to carry out dynamic simulations to obtain more realistic results, to estimate the RTE more accurately and to derive relevant ways of improving the process. Several studies suggest parametric analysis of AACAES RTE such as Guo et al. that focused on the throttle-valve pressure, or the number of compression and expansion stages [15], or Hartmann et al. that tested several AACAES configurations and process scenario [16]. Nevertheless, fewer studies address the full dynamical behavior of those processes. Peng et al. studied dynamic performances of a simple AACAES system but focused on packed bed design optimization and did not consider the volume storage transient behavior [17]. Barbour et al. investigated the transient behavior of packed bed TES but considering isothermal cavern. Sciacovelly et al. used a different approach by integrating the turbomachinery maps for transient studies [18] as well as a transient cavern model. Although only one stage of compression with one packed bed TES and a constant expansion ratio is simulated, the results confirm the importance of taking turbomachinery dynamics into account. Still with a dynamic approach Luo et al., studied a three-stage compression system but with indirect heat transfer [19] which stabilizes the turbomachinery inputs.

This work provides a simulation framework to investigate the performances of multiple AACAES systems under transient conditions. Section 2 presents the numerical model of each component of the framework. In section 3 a direct heat transfer AACAES type is studied, made of several packed beds with a porous solid media, which store at a higher temperature, but which has a greater effect on volume flow variations since its temperature of storage may vary with time. In section 4, an indirect heat exchange type consisting in pressurized water at a lower temperature but with constant temperature heat restoration is studied. Both simulation cases and their variants are designed to ensure that the variations in volume flow do not exceed 10 % of their average value so that the turbomachinery operates under manageable conditions around the maximum efficiency.

**Table:** Nomenclature

$T_{in}$	Inlet temperature (K)	$\eta_{T}$	Turbine polytropic efficiency (−)
$T_{out}$	Outlet temperature (K)	$\eta_{c}$	Compressor polytropic efficiency (−)
$P_{in}$	Inlet pressure (bar)	$\eta_{c,mech}$	Compressor mechanical efficiency (−)
$P_{out}$	Outlet pressure (bar)	$\eta_{c,elec}$	Compressor electrical efficiency (−)
$T_{\lim}$	Max output temperature (K)	$\eta_{t,mech}$	Turbine mechanical efficiency (−)
$\dot{m}$	Mass flow (kg.s^−1^)	$\eta_{t,elec}$	Turbine electrical efficiency (−)
$h_{in}$	Inlet specific enthalpy (J.kg^−1^)		
$h_{out}$	Outlet specific enthalpy (J.kg^−1^)	ρ	Air density (kg.m^−3^)
		γ	Heat capacity ratio
α	Air cooler coefficient (−)	μ	Air dynamic viscosity (kg.m^−1^.s^−1^)
$\Delta_{1}$	Temperature difference at heat exchanger output (K)	$C_{p}$	Specific heat capacity (J.kg^−1^.K^−1^)
$\Delta_{2}$	Temperature difference at heat exchanger input (K)		
ΔP	Pressure drop (bar)	u	Superficial velocity (m.s^−1^)
L	Packed bed depth (m)	⟨T⟩	Average temperature (K)
ε	Void fraction (−)	⟨k⟩	Average conductivity (W.m^−1^.K^−1^)
$A_{0}$	Specific area (m^−1^)	${\left\langle u\right\rangle }^{f}$	Average pore velocity (m.s^−1^)
d	Packed bed particles diameter (m)		
U	Heat exchanger heat transfer coefficient (W.m^−2^.K^−1^)	$P_{t}$	Thermal power (W)
		$P_{elec}$	Electrical power (W)
$h_{sf}$	Cavern heat transfer coefficient (W.m^−2^.K^−1^)	$P_{transfer}$	Exchanged heat duty (W)
$m_{cav}$	Cavern air mass (kg)		
$h^{+}$	Incoming air specific enthalpy (J.kg^−1^)	**Superscripts**	
$h_{cav}$	Outcoming air specific enthalpy (J.kg^−1^)	f	fluid
$dq_{a}$	Heat exchanged through cavern walls (J)	s	solid
$u_{cav}$	Internal air mass energy (J.kg^−1^)		

## 2 Modelling code the components

**Fig. 2:**
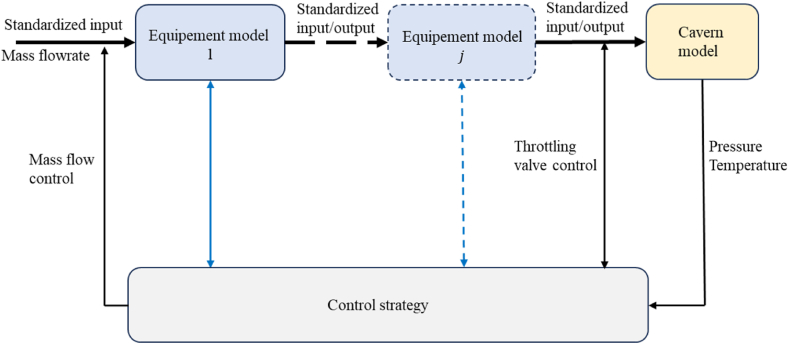
Principle of the structure of the code.

The model components are simulated using a numerical code written in Python 3. It is component-based and modular so that the components models can be improved and implemented easily (Fig. 2). Note that this tool is so modular that closed loop processes such as Carnot Batteries can also be simulated [20]. In order to simplify the process implementation inputs and outputs of each component have been standardized so that the output of one component can directly be re-used as input by others. Active control of some components can be activated according to specific criteria, such as heat exchange rate, temperature variations, electrical power, etc. So, the target value for one of the above criteria can be reached by output feedback control method modifying linearly, at each time step the mass flow, the outlet target pressure of turbomachinery, the air cooler target temperature etc. At this stage, only a design condition model is presented for compressors and turbines, but work is in progress to integrate off design consideration into our simulations. In the following sections, the most typical equipment models are described.

### 2.1 Compressors

In the code, compressors can be controlled either via target pressure strategy or via compression ratio strategy. Phase changes of the working fluid, such as water evaporation, are neglected. The compression process is polytropic and output temperature is related to the input temperature and compression ratio tanks to the polytropic efficiency coefficient $\eta_{c}$.

(1) $$T_{out}=T_{in}{\left(\frac{P_{out}}{P_{in}}\right)}^{\frac{\gamma -1}{\eta_{c}\gamma }}$$

Neglecting the variation of kinetic energy from inlet to outlet air during the compression lead to the following equation for the power consumption during the compression.

(2) $$P_{t}=\dot{m}\left(h_{out}-h_{in}\right)$$

Where $\dot{m}$ is the mass flowrate through the compressor and $h_{in}$ and $h_{out}$ respectively the inlet and outlet specific enthalpy.

The consumed electrical power $P_{elec}$ is related to the power $P_{t}$ via both the mechanical and electrical efficiencies $\eta_{c.mech}$ and $\eta_{c.elec}$.

(3) $$P_{elec}=\frac{P_{t}}{\eta_{c,mech}.\eta_{c,elec}}$$

### 2.2 Turbines

Following the same approach than for compressors, turbines expanders can also be controlled via target pressure or expanding ratio. Although the outside air taken contains humidity, the condensation associated with the lowering of the dew point during expansion is neglected in this study. And the expanding process considered as polytropic. The output temperature is related to the input temperature and expanding ratio thanks to the polytropic efficiency coefficient $\eta_{T}$.

(4) $$T_{out}=T_{in}{\left(\frac{P_{out}}{P_{in}}\right)}^{\eta_{T}\frac{\gamma -1}{\gamma }}$$

The power output is given by equation (2), the output electrical power $P_{elec}$ is related to the power $P_{t}$ via both the mechanical and electrical efficiencies $\eta_{t.mech}$ and $\eta_{t.elec}$.

(5) $$P_{elec}=-\eta_{t,mech}.\eta_{t,elec}{\;.P}_{t}$$

### 2.3 Air coolers

Air cooler operates via an air-air heat exchanger that uses the ambient air as coolant. The most common application is to reduce the compressor inlet temperature to prevent excessive outlet temperatures. It can also be used to cool the air before its storage in cavern or to control the performance of turbomachinery by stabilizing the temperature.

In this code, the air cooler is temperature controlled.

(6) $$\left\{ \begin{array}{c} T_{out}=T_{in}\;if\;T_{in}<T_{\lim}\\ T_{out}=T_{\lim}\;else\; \end{array}\right.$$

Pressure losses are either defined as constant input data or calculated as a function of a pressure loss coefficient varying with the square of the flow rate. The thermal dissipated power $P_{t}$ is calculated by an energy balance as in (2). The electrical power required to circulate the cooling ambient air in the heat exchanger is calculated using design rules commonly used in the process industry [21]. It depends on the outside temperature and the power to be dissipated.

(7) $$P_{elec}=-\alpha \frac{P_{t}}{LMTD\left(\Delta_{1},\Delta_{2}\right)}$$

Where $\Delta_{1},\Delta_{2}$ are the temperature differences at both end of the air cooler heat exchanger, LMTD is the Logarithmic Mean Temperature Difference defined as $LMTD\left(\Delta_{1},\Delta_{2}\right)=\left(\Delta_{1}-\Delta_{2}\right)/\left(\ln\left(\Delta_{1}\right)-\ln\left(\Delta_{2}\right)\right)$ and α is a coefficient defined empirically by the choice of air cooler type.

### 2.4 Thermal energy storage (TES)

#### 2.4.1 Packed bed energy storage: direct air contact

**Fig. 3:**
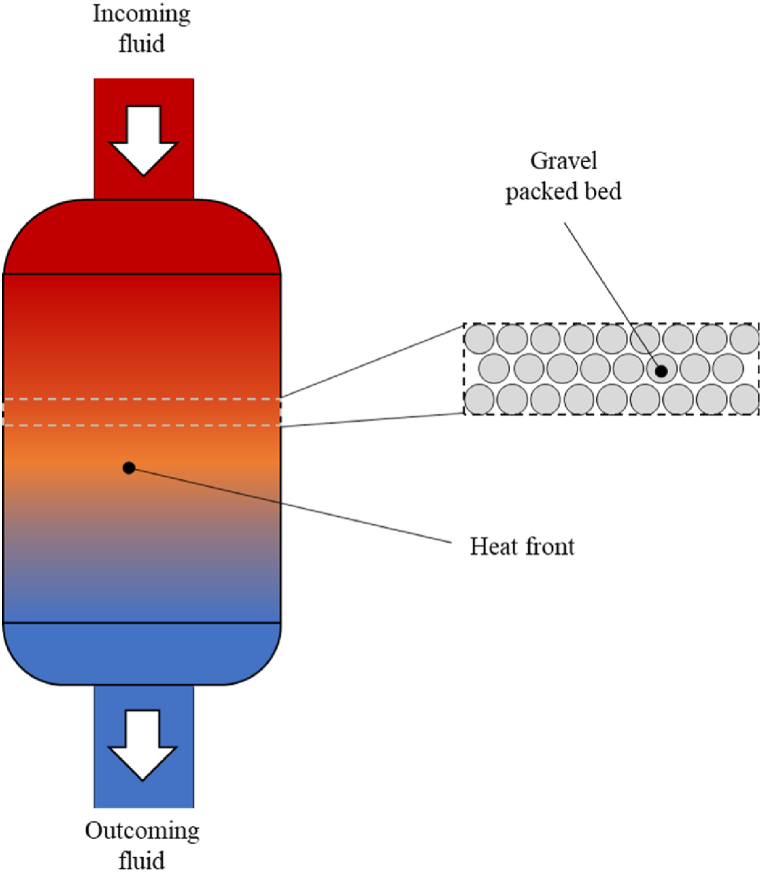
Schematic diagram of a packed bed TES.

During charging or discharging phase, the working fluid passes directly through the packed bed and transfers heat to the solid media (Fig. 3). This heat storage system is already a mature technology and is widely used to store thermal energy for various processes such as district heating, concentrated solar power, Brayton cycles or AACAES [[22], [23], [24]]. It has the advantage of being compact and withstanding a very large range of temperatures.

In the AACAES process, it is used to store the thermal energy generated by the compressors. There can be a packed bed dedicated to each compression stage or a common packed bed that mutualizes the thermal energy coming from all the compressors.

Packed bed energy storage is described by two equations, one for the solid side (subscript s) and the other for the fluid side (subscript f). In the literature [[25], [26], [27]], it is commonly assumed that the heat transfer is mono-dimensional, along the packed bed main flow axis. This model is called 1-dimensional Darcean flow model and it is described in Ref. [28] as continuous solid model.

(8) $$\frac{\partial {\left\langle T\right\rangle }^{f}}{\partial t}+{\left\langle u\right\rangle }^{f}\frac{\partial {\left\langle T\right\rangle }^{f}}{\partial x}=\frac{{\left\langle k\right\rangle }^{f}}{\varepsilon {\left(\rho C_{p}\right)}^{f}}\frac{\partial ²{\left\langle T\right\rangle }^{f}}{\partial ²x}+\frac{h_{sf}A_{0}}{\varepsilon {\left(\rho C_{p}\right)}^{f}}\left({\left\langle T\right\rangle }^{s}-{\left\langle T\right\rangle }^{f}\right)$$

And

(9) $$\frac{\partial {\left\langle T\right\rangle }^{s}}{\partial t}=\frac{{\left\langle k\right\rangle }^{s}}{\left(1-\varepsilon \right){\left(\rho C_{p}\right)}^{s}}\frac{\partial ²{\left\langle T\right\rangle }^{s}}{\partial ²x}-\frac{h_{sf}A_{0}}{\left(1-\varepsilon \right){\left(\rho C_{p}\right)}^{s}}\left({\left\langle T\right\rangle }^{s}-{\left\langle T\right\rangle }^{f}\right)$$

Where:

${\left\langle T\right\rangle }^{f}$ is the section average fluid temperature

${\left\langle T\right\rangle }^{s}$ is the section average solid temperature

$A_{0}$ is the specific surface area

${\left\langle u\right\rangle }^{f}$ is the average pore velocity

${\left\langle k\right\rangle }^{f}$ and ${\left\langle k\right\rangle }^{s}$ are the average conductivity of the fluid and solid derived from the Krupiczka model [29]

ε is the void fraction

$h_{sf}$ is the heat transfer coefficient that is estimated thanks to the Nusselt law described in [30]

Pressure loss through the packed bed is estimated using the Ergun’s equation [31].

(10) $$\frac{\Delta P}{L}=150.0\frac{\mu }{d^{2}}\frac{{\left(1-\varepsilon \right)}^{2}}{\varepsilon^{3}}u+1.75\frac{\rho }{d}\frac{1-\varepsilon }{\varepsilon^{3}}u^{2}$$

Where:

ΔP is the pressure drop

L is the packed bed thickness

d is the equivalent diameter of the packed bed particles

ρ is the density of the working fluid

μ is the dynamic viscosity of the working fluid

$u={\varepsilon \left\langle u\right\rangle }^{f}$ is the superficial velocity

The Ergun’s equation is valid, provided the bed is composed of similarly sized spherical particles. In practice, for reasons of cost, large particle beds are made up of non-spherical gravel. In this case, Ergun's relation tends to underestimate the pressure losses, [32], but by a sufficiently small order of magnitude (around 10 %).

**Fig. 4:**
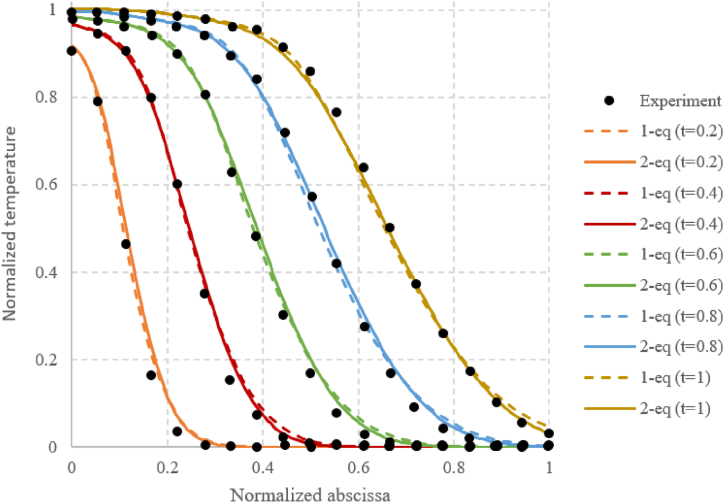
Validation case for the 2 equations and the 1 equation model. Time t is also normalized according to the duration of the charging phase.

This packed bed model has been validated on the Cascetta experiment described in Refs. [33,34] and showed good results since the heat front progresses in a similar way (Fig. 4).

All the packed beds considered in this paper are composed of air with low flow velocities and small solid spheres. The air conductivity and heat capacity are very small compared to those of the solid. Under these conditions, the heat exchange between the fluid and the solid is fast and it is relevant to consider a single-phase model with only one equation governing the average porous media, which is much faster to run. This model for gas-solid packed beds has been discussed in Refs. [35,36] and more recently in Refs. [23,37]. It assumes that the temperature of the fluid and the solid equalize within one time step. The temperature equations (8), (9) then become (11).

(11) $$\frac{\partial T}{\partial t}+u\frac{\partial T}{\partial x}=\left(\frac{{\left\langle k\right\rangle }^{f}}{\varepsilon {\left(\rho C_{p}\right)}^{f}}-\frac{{\left\langle k\right\rangle }^{s}}{\left(1-\varepsilon \right){\left(\rho C_{p}\right)}^{s}}\right)\frac{\partial ²T}{\partial ²x}$$

It has been validated on the same case than the two-equation model (Fig. 4) and showed good results.

Direct heat storage in packed bed, where the compressed air circulates directly in the bed, is advantageous because it limits heat losses. But it becomes technically challenging and economically irrelevant for large volumes and high pressure of storage. To overcome this limitation, the heat can be transferred via a heat exchanger to a coolant fluid circulating at lower pressure.

#### 2.4.2 Indirect heat transfer

In some cases, it may be relevant to retrieve stored thermal energy at a constant temperature. Using reservoirs of fluid avoids the problem of the thermal front that can occur in packed beds application for example.

**Fig. 5:**
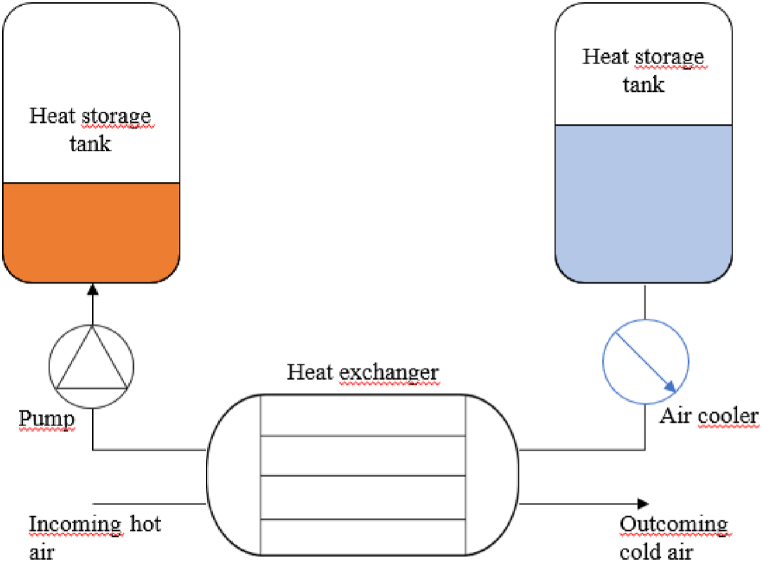
Schematic diagram of an indirect heat transfer assembly.

In this process, the heat is transferred to a specific heat exchange fluid via a heat exchanger. This fluid is transferred from one reservoir to another depending on whether heat needs to be extracted or released (Fig. 5). Depending on the coolant, the tank pressure is adapted so that there is no phase change. An air cooler could also be useful to control the outcoming air temperature.

Pressure losses in the air circuit have a direct impact on the overall efficiency of the AACAES. In order to limit the outcoming air temperature and minimize the impact on pressure drop, the air cooler is positioned on the coolant circuit. The trade-off is a slightly more complex control policy since the air cooler is not acting directly on the working fluid (Fig. 5). During the discharging phase, this air cooler is not activated.

The disadvantage of these systems is that they require more equipment and more control. Especially when the outlet temperature needs to be precisely controlled while the inlet conditions vary. However, these are all mature technologies that are already widely used in processes.

The assembly is modelled as a single component for which an energy balance system is solved at each iteration. The first equation is the Logarithmic Mean Temperature Difference equation (12) with a constant heat transfer coefficient U and surface A. As we neglect all thermal losses in the heat exchanger, we can equalize the transferred thermal power (13). Solving these equations system provides, the coolant fluid flow rate, the outlet pinch temperature and the tank filling temperature. It should be noted that assuming a constant exchange coefficient is questionable as inlet temperature and volume flow rate are expected to vary with time, and that work is underway to better model the heat transfer.

(12) $$P_{transfer}=U.A.LMTD\left(\Delta_{1},\Delta_{2}\right)$$

(13) $${\dot{m}}_{cool}\left(h_{cool,out}-h_{cool,in}\right)=-{\dot{m}}_{work}\left(h_{work,out}-h_{work,in}\right)$$

It may happen at the end of the phase that a little liquid remains in one of the tanks. To balance the cycles, this residue is added to the other tank so that one is empty and the other full. In practice, the tanks are designed so that this residue is negligible.

### 2.5 Air reservoir

Most projects in operation or under serious investigation use geological caverns with a fixed volume. Therefore, the amount of air loaded or unloaded during the charging and discharging phase leads to variations in pressure and temperature, which can affect the volume flow, and consequently, the efficiency of the turbomachinery and the RTE. In this part we describe the dynamical model used by our simulation code.

During the storing phase, the incoming internal energy and the pressure work is added to the existing internal energy. According to the first law of thermodynamics for open systems and neglecting the kinetic and the potential energy of the air, the equation of the cavern is given by (14).

(14) $$d\left(m_{cav}u_{cav}\right)=h^{+}dm_{cav}+dq_{a}$$

Where.

$m_{cav}$ is the mass of air into the cavern

$u_{cav}$ is the specific internal energy of the air into the cavern

$h^{+}$ is the incoming specific enthalpy to the cavern from outside

$dm_{cav}$ is the mass iteration coming into the cavern

$dq_{a}$ is the heat exchanged with the cavern walls

During the discharging phase, the outcoming specific internal energy is those of the cavern.

(15) $$d\left(m_{cav}u_{cav}\right)=h_{cav}dm_{cav}+dq_{a}$$

Where.

$h_{cav}$ is the outcoming specific enthalpy from the cavern to the outside.

**Fig. 6:**
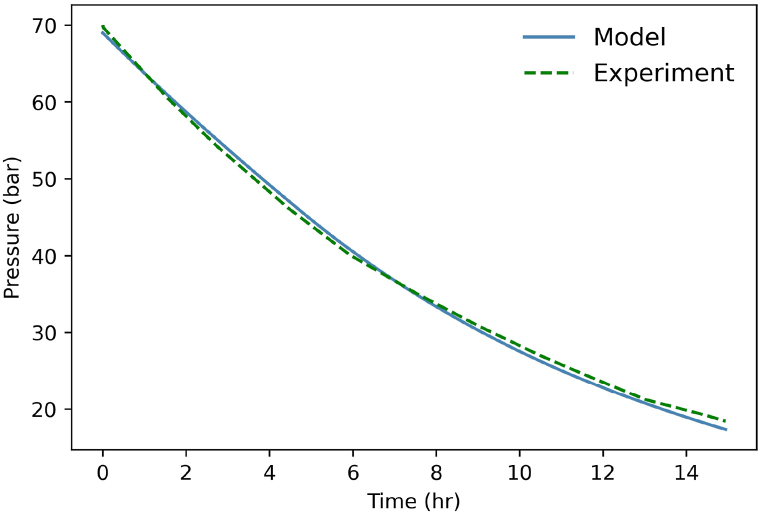
Pressure into the cavern during deep discharge. Comparison between Huntorf case and model.

**Fig. 7:**
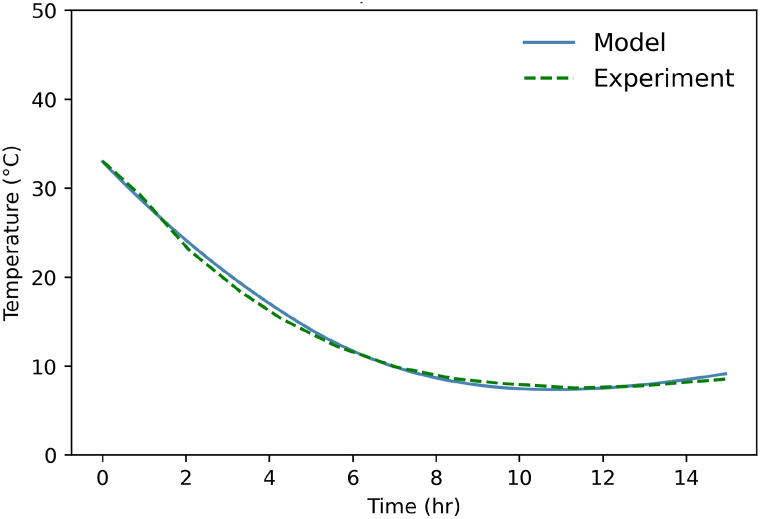
Temperature into the cavern during deep discharge. Comparison between Huntorf case and model.

The numerical model is validated on the Huntorf experimental case (Fig. 6, Fig. 7) depicted in Ref. [38]. The heat exchange with the wall, which is essential for this comparison [39], has been evaluated using the method described in Ref. [39].

In practice, the method of calculating the heat exchange coefficient is specific to the Huntorf cavern and there would be little point in using it for other simulations for which the wall properties are unknown. Furthermore, in this paper the cavern will operate at a higher pressure and with less variation leading to less temperature variations. We will therefore restrict ourselves to an adiabatic model.

### 2.6 Throttling valve

**Fig. 8:**
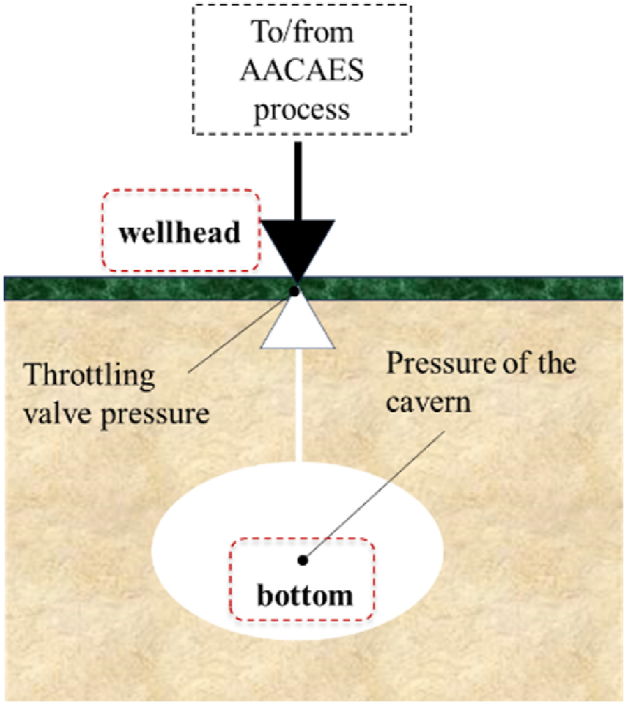
Schematic view of an underground cavern and the throttling valve.

Depending on the volume of the cavern and the duration of the phases, the maximum and minimum pressure amplitude inside the cavern may vary widely, implying large volume flow variations and preventing turbomachine operation. To limit those variations and to use the turbomachinery close to its design conditions, a throttling valve at the cavern inlet controls the compression or expansion pressure (Fig. 8). In our model, the throttling process is isenthalpic so that there is a negligible change in temperature.

Note that there may be a difference between the throttling valve target pressure at wellhead and the effective cavern pressure at bottom because of the static air pressure column and to pipe pressure loss. This value depends on the depth of the cavern and the pressure and temperature at the throttling valve.

### 2.7 Validation summary

Packed bed and cavern models have been validated with real applications (2.4.1, 2.5), while the other components such as air coolers, heat exchangers, pumps, etc. are well known models already used in several process software.

## 3 AACAES process

There are several variations of the AACAES process, depending on storage pressure, storage temperature and the duration of the charge and discharge phases. In this paper we will investigate two case studies systems with a 100 MW electricity power and a storage capacity of 400 MWh. To achieve this level of performance, multiple compression stages are required to progressively remove the compression heat and avoid temperatures that are unbearable for turbomachinery. Note that in most papers where multiple compression stages are studied, a single TES pools the heat from and to the turbomachinery [10,14,15,19]. In our case, we preferred to associate a system with each compressor or turbine, as this maximizes the heat extracted, since each turbomachine does not necessarily have the same inlet and outlet temperatures.

Both case studies use different types of thermal energy storage so that the comparison highlights their advantages and disadvantages in terms of volume flow variation under transient conditions. The first uses direct storage systems via packed beds at high temperatures (above 230 °C), but the volume flow variations, inherent in these systems, limit their sizing. The second, by comparison, will use indirect heat transfer and thermal storage with water at low temperature and low pressure. The lower temperature storage will penalize the RTE, but the use of water will also have the advantage of reducing cost. In any case, the thermodynamic of rest phases is neglected to simplify the comparison and to avoid introducing unknown parameters such as heat exchange coefficients with the outside, conductivity of the tank walls, etc. More generally, heat loss can be neglected if good insulation is provided in accordance to Refs. [25,40].

Note that the case studies are designed so that the maximum flow variation never exceeds 10 %. Under these conditions, the efficiency of the compressors and turbines is assumed to be constant, since only minimal control measures are required to maintain their performance under these conditions.

### 3.1 Packed bed heat storage: direct air contact

**Fig. 9:**
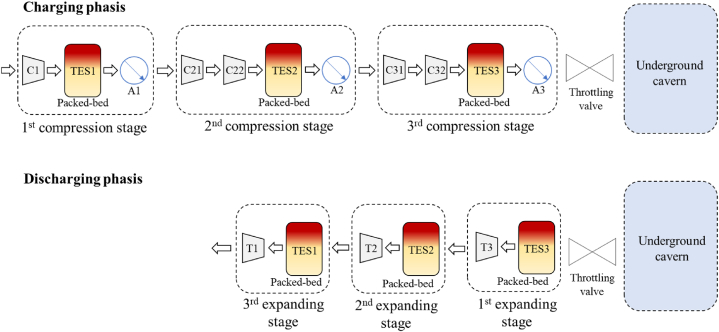
Schematic diagram of the direct air contact process and its equipment for charging and discharging phases.

In this example, the AACAES operates with three compression and expansion stages (Fig. 9). The throttling valve is controlled so that the outlet compressors pressure during the charging phase and the inlet turbine pressure during the discharging phase remains constant. The throttling valve is essential for stabilizing the target pressure of the compressors and the inlet pressure of the turbines. Without it, variations in cavern pressure would cause the turbomachines to operate completely outside their operating range.

The solid media used in the packed bed consists of various gravels whose heat capacity as a function of temperature, porosity and equivalent diameter have been experimentally estimated in our laboratory at IFP Energies Nouvelles.

The choice of turbomachinery operating pressures, the number of compressors and turbines and their efficiencies are realistic assumptions given the usual constraints of manufacturers we contacted. The outlet temperature of TES systems may increase at the end of the load phase due to thermal overloading, to the point of exceeding the allowable inlet temperature of the next compressor. In this case, air coolers are activated to remove the excess heat before it enters the compressors. The performance and characteristics of the air coolers are based on the expertise of our Chemical Process department. Finally, TES are designed to minimize the use of air coolers, which dissipate heat and have a negative impact on efficiency, bearing in mind that the larger TES are, the more expensive they are.

The main parameters of the process are depicted in Table 1.

**Table 1:** Overall process design of the AACAES with packed beds (nominal case).

Turbomachinery			
		Nominal target pressure	Isentropic efficiency

C1	Gas compressor	5.1 bar	0.85
C21	Gas compressor	10.2 bar	0.85
C22	Gas compressor	23.5 bar	0.85
C31	Gas compressor	50.4 bar	0.85
C32	Gas compressor	113.3 bar	0.85
T1	Turbine	Atmospheric pressure	0.89
T2	Turbine	5.0 bar	0.89
T3	Turbine	23.6 bar	0.89

Air cooler			

A1, A2, A3	Air cooler	Max working fluid temperature: 50 °C	
		Pressure loss at nominal flowrate: 300 mbar	
		α coefficient: 0.901	
TES – packed bed			
Porosity: 0.35			
Particle diameter: 0.01 m			
Particle specific heat capacity (J.kg^−1^.K^−1^): $Cp\left(T\right)=4.9+3.2T\;–\;2.3e^{-3}\;T^{2}$			
		Working fluid	Total volume
TES1	Packed bed TES	Air	1930 m^3^
TES2	Packed bed TES	Air	1865 m^3^
TES3	Packed bed TES	Air	2020 m^3^

Cavern			

Underground cavern	Volume: 170 000m^3^		
	Min working pressure: 110.0 bar		
	Max working pressure: 131.8 bar		
	Cavern depth: 1 km		
	Gravity loss: 10 bar		
	Pressure loss during charging phase: 1.0 bar		
	Pressure loss during discharging phase: 2.0 bar		
Regulation			

Throttling valve	Charging pressure: 123.0 bar		
	Discharging pressure: 98.0 bar		
Nominal mass flow			

Load: 140 kg/s			
Unload: 200 kg/s			

#### 3.1.1 Steady state

The steady state is reached after 30 cycles starting from ambient conditions, with a homogeneous packed bed at 27 °C and an initial cavern pressure of 110 bar, corresponding to its minimum operating point. In this state, the cycles are periodic, and the influence of the initial conditions has been mitigated.

**Fig. 10:**
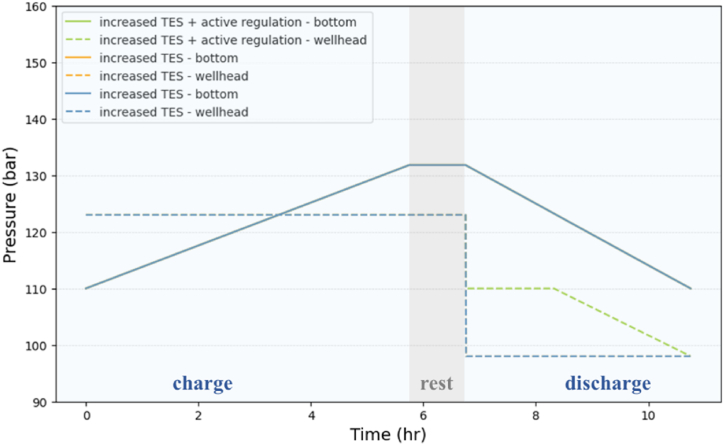
Pressure into the cavern (solid line) and throttling valve upstream pressure during charging and downstream pressure during discharging (dotted line). “nominal” and “increased TES” are superimposed.

The charging phase begins with the internal cavern at its minimum operating pressure of 110 bar. It takes 5 h and 46 min to reach the maximum pressure of 131.8 bar. During this phase, the pressure in the cavern increases but the throttling valve maintains a constant pressure at wellhead (Fig. 10). This allows the compressors to work optimally at constant outlet pressure. The discharge is shorter and takes 4 h to return to the initial pressure. During discharge, the throttling valve ensures a constant inlet pressure to the turbines.

**Fig. 11:**
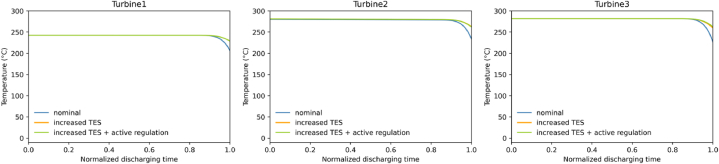
Inlet temperature into turbine during discharging phase, “increased TES” and “increased TES + active regulation” are superimposed.

**Fig. 12:**
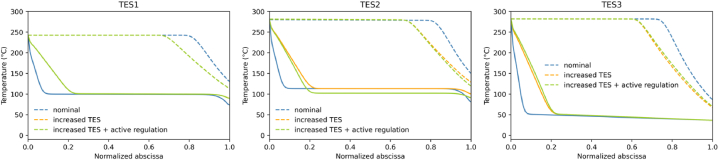
TES temperature profiles at the start (dash line) and at the end of discharging phase (solid line). For TES1, “increased TES” and “increased TES + active regulation” are superimposed.

However, during the last 40 min, the heat returned by the packed beds begins to be insufficient and the outlet temperature drops (Fig. 11, Fig. 12). This leads to a variation in volume flow in the turbomachinery and a drop in the retrieved electrical power.

For this nominal design the packed beds volumes had been minimized so that the maximum volume flow variations do not exceed 10 % (Table 2). In this case, the heat storage volume is the limiting factor.

**Table 2:** Maximum volume flow variations during the discharging phase and RTE.

Turbomachinery	Case 1	Case 2	Case 3
	Nominal	Increased TES	Increased TES + Active regulation
T1	9.14 %	4.56 %	10.28 %
T2	8.84 %	3.43 %	9.46 %
T3	10.40 %	4.09 %	9.51 %
RTE	**69.9 %**	**69.8 %**	**70.9 %**

A second variant labeled “increased TES” which increases the length of each packed bed in order to get 20 % more storage volume, is investigated. This is expected to increase heat storage capacity and thus compensate for the heat deficit, and temperature drop, at the end of the discharge. The simulation shows that it reduces the volume flow variations to 5 % maximum (Table 2). However, it should be noted that increasing the volume of the packed beds by 20 % is not sufficient to avoid temperature drop at the end of the discharge. This is due to the cumulative effect of the temperature dispersion phenomenon [26,27,40] leading to a gradual flattening of the heat front from cycle to cycle.

Furthermore, the RTE is not improved as expected (Table 2). Firstly, because in both cases, the temperature drop is small and only occurs in the last tenth of the discharge, so the expected gain is limited anyway. But in addition, adding 20 % of packed beds volume results in a slight increase in pressure drop (a few hundred mbar) which has a negative impact on the RTE and compensates for the temperature gain.

Another option could be to take advantage of the improved volume flow stability of the “increased TES” variant to slightly relax the pressure constraint on discharge regulation to allow more variations in volume flow. This second variant labeled “increased TES + active regulation” now imposes a maximum discharging pressure on the downstream side of the throttling valve of 110 bar instead of 98 bar in the first two cases (Fig. 10). This inactivates the throttling valve around the mid-discharge point and from there the cavern pressure variations have a direct effect on the inlet turbine pressure variations. This updated pressure threshold is chosen so that the maximum volume flow variation remains about 10 % (Table 2). In this configuration, we see that, with the throttle valve inactive, the difference between bottom and wellhead pressure is 12 bar (Fig. 10), which correspond to the 10 bar of gravity loss plus 2 bar of pressure loss (Table 1).

This result in an increased efficiency but with higher volume flow variations since the effect of pressure variations on the downstream side of the throttling valve is now added to the outlet temperature variations of packed beds. Indeed, in the first part of the discharge, pressure and temperature are constant, so density and therefore volume flow are constant for a constant mass flow. Then, the temperature remains constant but the pressure decreases, inducing a decrease in density and consequently an increase in volume flow for a constant mass flow. Finally, particularly at the end of the discharge, the temperature drops, which increases the density and therefore decreases the volume flow (for a constant mass flow), but this is partly offset by the decrease in pressure, which tends to decrease the density.

It is important to note that the derived efficiencies do not consider the variable performance of the turbomachinery as a result of variations in volume flow. Although it has been shown to be predominant in some conditions, such as transient start-up and shut-down phases [40,41]. It would therefore be irrelevant to draw conclusions about the performance of one case compared to another with such small differences in RTE. Nevertheless, this comparison allows us to observe trends as a function of different design policies.

This study focuses on a rarely studied aspect: the impact of TES design and cavern connection on volume flow variation which, at the end, has an impact on turbomachinery operation. Some literature discusses the variation of efficiency as a function of reduced flow rate, but this often considers a very basic control strategy that leads to conclusions that are difficult to interpret. In our studies, we have simplified the turbomachinery mappings so as not to bias the observed results with an additional degree of freedom. By integrating the efficiency maps for the turbomachinery and a proper optimized control policy, the case with larger TES would certainly have higher performances.

#### 3.1.2 Energy balance

Table 3, details the energy balance in the components for the different cases. We can see by comparing Case 1 and Case 2, that the amount of heat stored in the TES is independent of its volume. As stated in (3.1.1), increasing the volume of the TES by 20 % only allowed to store 1 % more heat. Indeed, an analysis of Fig. 12 shows that increasing the length of the TES modifies the temperature profile in the TES. The temperature fronts are less stiff. In addition, the zones close to the TES inlet and outlet are less involved in heat storage. The increased TES has found its equilibrium to store the same amount of energy. The energy stored does not change, as it is partly controlled by the amount of air stored in the cavity. In fact, it is pressurized air that converts heat into work in the turbines. If there's no more air, the system stops, and no more heat is consumed. Between Case1 and Case2, since the flow rates and durations are the same, the amount of heat involved is the same.

**Table 3:** Absolute energy balance during charge and discharge detailed by equipment (MWh).

	Case 1	Case 2	Case 3
**Charging phase**			
**Compressor 1**	176.9	176.9	176.9
**TES1**	117.5	117.0	117.1
**Air cooler 1**	40.6	41.0	41.0
**Compressor 21**	79.4	80.1	80.1
**Compressor 22**	109.7	109.9	109.9
**TES2**	136.7	137.9	146.8
**Air cooler 2**	52.0	51.7	42.7
**Compressor 31**	81.3	81.3	81.3
**Compressor 32**	109.9	109.9	109.9
**TES3**	190.4	191.3	191.2
**Air cooler 3**	1.6	0.7	0.8
**Discharging phase**			
**Turbine 1**	136.5	135.8	133.5
**TES 1**	117.4	116.9	116.9
**Turbine 2**	148.3	148.8	148.8
**TES2**	136.6	137.7	146.7
**Turbine 3**	139.0	139.3	148.4
**TES 3**	190.4	191.4	191.2

The 1 % variation in stored heat is due to the fact that the first TES was too short (Fig. 12) and part of the temperature front reached the TES outlet. This problem is mitigated with the extended TES.

On the other hand, in Case 3, there is a higher pressure at the beginning of discharge (Fig. 10) which allows more energy to be recovered via Turbine 3, but also results in a lower turbine outlet temperature. This lower temperature has an effect in the TES2, and the mean temperature profile at the end of discharge is lower (Fig. 12). This improves the energy storage capacity of TES2 over the cycles (Table 3) by about 10 % compared to Case 1, the same case but with constant inlet pressure. Contrary to intuition, the storage capacity of TES2 has increased more by adjusting the valve than by increasing the volume.

More precisely, the heat extracted from the air between compressor 22 and compressor 31 is partly stored in the TES 2 and partly dissipated by the air cooler 2. In Case 1, the TES2 is less cooled by the discharge (Turbine 3). This means less heat is stored in TES2 and more is extracted by the air cooler 2. In the Case 3, TES2 is more cooled by the discharge (Turbine 3). This means more heat is stored in TES2 and less is extracted by the air cooler 2. Overall, the sum of the amounts of heat stored in the TES2 and extracted by the air cooler 2 remains constant.

**Fig. 13:**
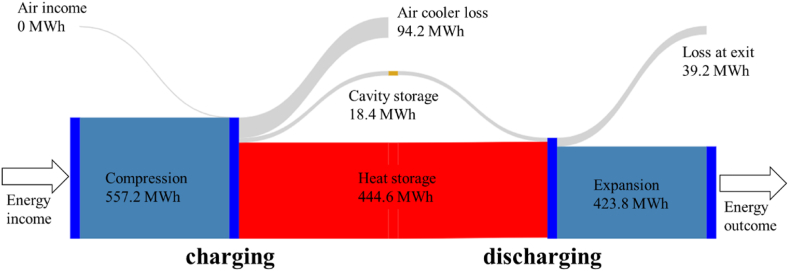
Energy flux summary over a cycle for packed bed heat storage (nominal case). The reference temperature is the outside temperature (27 °C).

Note that approximately 17 % of the compression energy is then dissipated through the air coolers and 7 % is released directly into the ambient air during the discharging phase. In fact, the air is returned at 75 °C, whereas the ambient air temperature is assumed to be 27 °C (Fig. 13).

### 3.2 Water based AACAES: indirect heat exchange

**Fig. 14:**
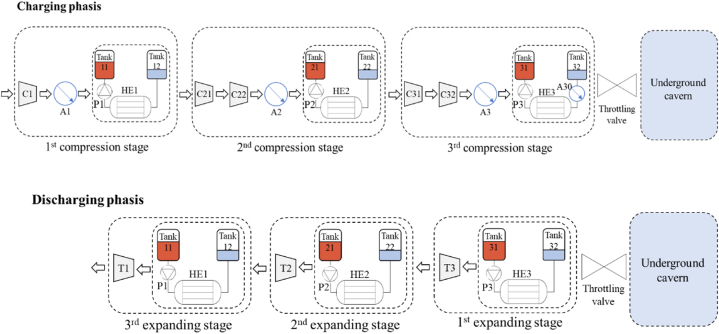
General diagram of the process and its equipment for charging and discharging phases.

To limit temperature variations, thus volume flow variations in turbomachinery, during heat restoration, packed beds are replaced by water tanks (Fig. 14). They are used in parallel circuits as heat storage media. The main advantage of using water tanks is that the heat is stored and restored at constant temperature.

The use of water as a heat transfer fluid and storage medium reduces costs but limits the storage temperature given the pressure constraints to keep the water in the liquid phase. In this case, the tanks are pressurized, and the water is stored at 6 bar, so that it can reach a maximum temperature of 150 °C without boiling. The downside of this low-pressure solution is that air coolers must be installed upstream of the storage systems to remove some of the heat from the compression, to prevent the risk of the water boiling.

Moreover, the three heat exchangers, HE1, HE2 and HE3, increase pressure losses throughout the circuit, on the air side, and have a direct impact on the RTE.

To simplify the comparison with the packed bed storage depicted above, the target performance of this process remains 100MW/400 MWh. The turbomachinery parameters such as efficiency, target pressure etc for charging and discharging phase are the same as in 3.1. In this process, air coolers are used to cool down the compressed air before it enters the heat exchangers to prevent water boiling. Their target temperature is 155 °C to account for 5 °C of pinch in the heat exchangers. These air coolers waste a lot of the heat generated by the compression which could be mitigated by increasing the storage pressure of the water and raising its boiling point. This solution would be more expensive as the tanks would have to be designed for higher pressure.

During the charging phase, an additional air cooler on the 3rd compression stage assembly is added to prevent air temperature to rise higher than 50 °C at the entrance of the cavern. Indeed, avoiding high cavern temperature enables to store more air since the pressure variations will be lower. The tanks are optimally designed to minimize the amount of water remaining to reduce the temperature drop when the fluids are recombined at the end of each phase (2.4.2). The heat exchanger surfaces have been iteratively designed to match the tank fluid volume and to maintain a 5 °C pinch at the working fluid inlet.

The element parameters are summarized in Table 4.

**Table 4:** Overall process design of the AACAES with water tanks (nominal case).

Turbomachinery			
		Nominal target pressure	Isentropic efficiency

C1	Gas compressor	5.1 bar	0.85
C21	Gas compressor	10.2 bar	0.85
C22	Gas compressor	23.5 bar	0.85
C31	Gas compressor	50.4 bar	0.85
C32	Gas compressor	113.3 bar	0.85
T1	Turbine	Atmospheric pressure	0.89
T2	Turbine	5.0 bar	0.89
T3	Turbine	23.6 bar	0.89

Air cooler			

A1, A2, A3	Air cooler	Max working fluid temperature: 155 °C	
		Pressure loss at nominal flowrate: 300 mbar	
		α coefficient: 0.901	
A30	Air cooler	Max working fluid temperature: controlled so that cavern inlet is 50 °C	
		Pressure loss at nominal flowrate: 300 mbar	
		α coefficient: 0.901	
Heat exchanger			
		Surface	Pressure loss at nominal flowrate

HE1, HE2, HE3	Heat Exchanger	12500 m^2^	300 mbar

TES – water tank			
		Working pressure	Water mass

TES1	Water tank	6.0 bar	950 ton
TES2	Water tank	6.0 bar	950 ton
TES3	Water tank	6.0 bar	950 ton

Pump			

P1, P2, P3	Pump	Target pressure: 6.0 bar	

Cavern			

Underground cavern	Volume: 223 000m^3^		
	Min working pressure: 110.0 bar		
	Max working pressure: 131.8 bar		
	Cavern depth: 1 km		
	Gravity loss: 10 bar		
	Pressure loss during charging phase: 1.0 bar		
	Pressure loss during discharging phase: 2.0 bar		
Regulation			

Throttling valve	Charging pressure: 123.0 bar		
	Discharging pressure: 98.0 bar		
Nominal mass flow			

Load: 146 kg/s			
Unload: 258 kg/s			

#### 3.2.1 Steady state

**Fig. 15:**
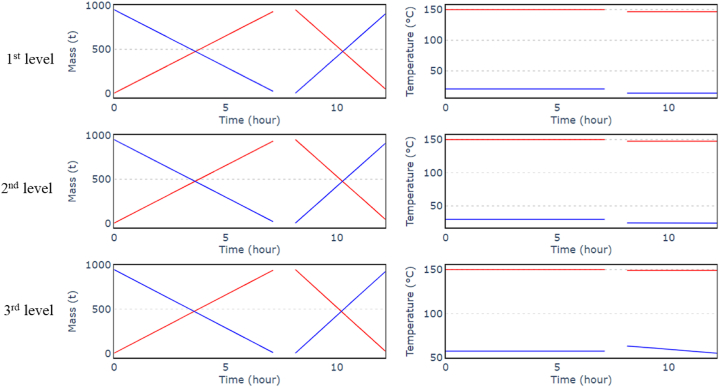
Mass (left) and temperature (right) variations with time for hot tank (in red) and cold tank (in blue) for the nominal case. (For interpretation of the references to colour in this figure legend, the reader is referred to the Web version of this article.)

The charging or discharging phase stops when the limit pressure in the cavern is reached (110.0 bar–131.8 bar). Therefore, the heat transfer may not be complete, and some unused fluid may remain in the emptied tank. To ensure that this fluid can be used during the next phases, it is added to the other tank fluid. As the amount of liquid is small, its temperature has a negligible effect on the storage temperature (Fig. 15). At the third level, the cold-water tank is filled with water at varying temperature due to the varying temperature into the cavern during the discharge. In both cases, a heat balance is carried out to calculate and impose the average temperature in the reservoir.

Simply replacing the packed beds with parallel water tanks reduced the variations in volume flow to 0 %. On the other hand, the lower storage temperature and the pressure losses generated by the heat exchangers reduced the efficiency to around 55 % (Table 5).

**Table 5:** Maximum volume flow variations during the discharging phase.

Turbomachinery	Case 1Case 2 - active regulation	
	nominal	Active regulation
T1	0 %	10.34 %
T2	0 %	9.82 %
T3	0 %	9.61 %
**RTE**	**55.4 %**	**56.6 %**

This improvement in volume flow variation theoretically leaves more room to control the cavern outlet pressure via the throttling valve. In this case, labeled as "active regulation", we impose a maximum discharging pressure on the downstream side of the throttling valve of 108 bar. This threshold is chosen so that the variation in volume flow rate does not exceed 10 % (Table 5). Although this configuration increases the expansion potential in the turbines, it only improves the RTE by 1 % (Table 5).

Compared to packed bed systems, the efficiency has decreased by 15 %. This means that less energy is stored for the same amount of air. Therefore, more air is needed to recover the same amount of energy. Note that although only the thermal storage systems were replaced, the cavern volume also had to be increased by 31 % to meet the 100MW/400 MWh discharge criteria.

#### 3.2.2 Energy balance

In the presence of water tanks, the lower RTE is mainly due to a much greater dissipation of energy by the air cooler systems to avoid overheating the water in the exchangers during the charging phase. Almost 43 % of the compression energy is dissipated by the air coolers. This leaves less thermal energy storage in the heat exchanger TES compared to the packed bed configuration (Table 6). Note that slightly more energy is stored in the cavern as it is larger for the water tank case.

**Table 6:** Absolute energy balance during charge and discharge detailed by equipment (MWh).

	Case 1	Case 2
**Charging phase**		
**Compressor 1**	228.1	228.1
**Air cooler 1**	93.4	93.4
**Heat exchanger 1**	140.3	141.1
**Compressor 21**	98.1	97.8
**Compressor 22**	129.9	129.5
**Air cooler 2**	87.7	86.3
**Heat exchanger 2**	130.4	139.4
**Compressor 31**	100.3	97.4
**Compressor 32**	133.4	129.4
**Air cooler 3**	103.3	87.4
**Heat exchanger 3**	110.7	110.7
**Air cooler 30**	9.0	13.4
**Discharging phase**		
**Turbine 1**	140.3	138.7
**Heat exchanger 1**	140.1	140.9
**Turbine 2**	141.7	141.2
**Heat exchanger 2**	130.2	139.2
**Turbine 3**	133.8	139.8
**Heat exchanger 3**	101.6	97.2

As with the packed bed, the pressure control results in cooler temperatures at the outlet of turbine 3 as the expansion rate is higher during discharge. Downstream, the TES2 compensates by providing the excess heat it was able to store during charging due to the lower stored water temperature. The restored power increase in case 2 is small, about 1 %, and is only effective at turbine 3.

**Fig. 16:**
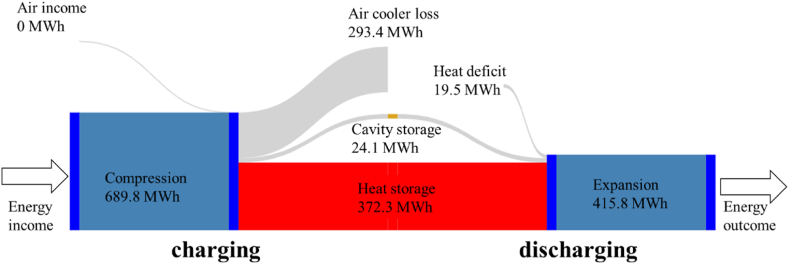
Energy flux summary over a cycle for water tank heat storage (nominal case). The reference temperature is the outside temperature (27 °C).

Note that since the exhaust air is at a lower temperature than the ambient temperature (27 °C), it effectively benefits from about 19.5 MWh of external heat over one cycle (Fig. 16).

## 4 Conclusion

It is crucial to consider volume flow variations during the charging and discharging phases as they have a huge impact on the operation of turbomachinery and hence, to the RTE. In this paper, we first analyze the effect of temperature management on volume flow variations. Then, we analyzed the effect of pressure management on volume flow variations. The two case studies in this paper have shown that the numerical tool used is relevant for estimating and comparing the performance of different AACAES systems.

We showed that, increasing the volume of the packed beds by 20 % only allowed 1 % more heat to be stored and therefore had little effect on mitigating the volume flow variations. This is due to the temperature dispersion phenomenon after several cycles.

On the other hand, indirect heat exchange solves this problem but has been shown to reduce RTE by requiring about 40 % of the heat generated by the compressors to be dissipated. This configuration also requires a 30 % increase in the volume of the cavern.

This could be improved with a higher working pressure and a stronger design. However, this may ultimately make indirect heat exchange less cost-effective. The use of pressure control of the throttling valve proves to be efficient in increasing the RTE, but this cannot be rigorously quantified as the gain is too small with respect to the modelling assumptions.

Although many key indicators are well covered by this numerical tool, the net RTE is more difficult to obtain, as it depends on turbomachinery operation, which requires a finer modelling than proposed. Simply implementing turbomachinery efficiency maps would not be meaningful because the process involves multiple compressors and turbines. A realistic model also necessitates a fully coupled control policy for the entire turbomachinery. New implementations are underway to add realistic compressor and turbine control so that the choice of charge and discharge phases pattern can be based on technical and economic criteria rather than simple pressure and temperature criteria.

## CRediT authorship contribution statement

**Victor Dupin:** Writing – review & editing, Writing – original draft, Validation, Software, Investigation, Conceptualization. **David Teixeira:** Writing – review & editing, Writing – original draft, Validation, Supervision, Methodology, Conceptualization.

## Declaration of competing interest

The authors declare that they have no known competing financial interests or personal relationships that could have appeared to influence the work reported in this paper.
